# Epidemiology and Control of Plant Diseases

**DOI:** 10.3390/plants12040793

**Published:** 2023-02-10

**Authors:** Alessandro Vitale

**Affiliations:** Department of Agriculture, Food and Environment, University of Catania, Via Santa Sofia, 100-95123 Catania, Italy; alevital@unict.it

I am pleased to present this edition of the Special Issue of Plants, dedicated to multifaceted topic of epidemiology and control of plant diseases in agricultural systems.

Regarding this point, it is noteworthy that some plant disease epidemics are currently having shocking effects on agricultural and forestry heritage and food crop production all over the world. Moreover, global climatic changes caused by human activities have progressively induced a significant increase of global mean temperature with direct repercussions on the epidemiology of plant pathogens. As a consequence, life cycles of plant pathogens could be deeply altered or an increase of emerging plant diseases could be observed on many commercial crops in different agroecosystems [1,2]. Thus, a better understanding of plant pathogen epidemiology, as well as individuation of new bio-based products, forecasting models, deep learning and reducing chemicals use approaches exploited for plant protection could better satisfy the development of ecofriendly, safer, and sustainable management strategies [3,4,5]. In light of above considerations, researchers and technicians are invited to investigate on epidemiological cycles of airborne and soilborne plant pathogens with the aim of ensuring good agricultural productivity, maintaining effective management of plant diseases and, simultaneously, good economic and environmental sustainability. This Special Issue underlines the performances of new eco-sustainable means of control to increase knowledge for plant pathologists, international scientific communities, and industries, and will provide a better understanding of the mode of action and timing application procedure of control means in different soil–plant systems. At the same time, an early and deep understanding of plant disease epidemiology is needed to tackle future challenges ahead and to relate directly with the disease control strategies.

Comprehensively, the Special Issue collected 13 original contributions (1 review, 1 perspective, and 11 research papers).

The review of Makhumbila et al. [6] provides a progress update on metabolomic studies of legumes in response to different biotic stresses. In their contribution, metabolome annotation and data analysis platforms are discussed together with future perspectives. The integration of metabolomics with other “omics” tools in breeding programs can aid greatly in ensuring food security through the production of stress tolerant cultivars.

In a perspective article, Figlan et al. [7] highlights the importance of breeding tools for improving resistance to Fusarium head blight disease caused by *Fusarium graminearum* in wheat as well as for limiting mycotoxin contamination to reflect the current state of affairs. According to the outlined scenario, only by combining these aspects in wheat research and development will it be possible to promote sustainable quality grain production and safeguard human and livestock health from mycotoxicoses.

In their research article, Thomidis et al. [8] compare two models, a generic and a polynomial model, respectively, for the forecasting in field appearance of olive leaf spot caused by *Venturia oleaginea*. The results obtained with both models correctly predicted infection periods, although differences regarding the severity parameter were reported according to the goodness-of-fit for the data collected on olive leaves in 2016, 2017, and 2018. Specifically, the generic model predicted lower severity values, which fits well with the incidence of the disease symptoms on unsprayed trees. Otherwise, the polynomial model predicted high severity levels of infection, but these did not fit well with the incidence of disease symptoms. This paper also establishes that a temperature within 5–25 °C range was appropriate for conidial germination, being 20 °C the optimum. At the latter temperature, it is also found that at least 12 h duration of leaf wetness is needed to start the germination of *V. oleaginea* conidia.

Similarly, Ji et al. [9] also investigate the effects of temperature and wetness duration on the infection severity of *Coniella diplodiella,* causal agent of grapevine white rot, by using artificial inoculation of grape berries through via infection pathways (uninjured and injured berries, and through pedicels). Results show that injured berries were affected sooner than uninjured ones, and disease increased as the inoculum dose increased. Irrespective of the infection pathway, 1 h of wetness is sufficient to cause infection at any temperature tested (10–35 °C), with the optimal temperature being 23.8 °C. The length of incubation is shorter for injured berries than for uninjured ones, and it is shorter at 25–35 °C than at lower temperatures. Finally, authors develop mathematical equations that fit well with the data, infection through any infection pathway, and incubation on injured berries, which could be used to predict infection period and, thus, to schedule fungicide applications.

The research paper of Verhoeven et al. [10] presents data on seed transmission of pospiviroids from large-scale grow-out trials of infested pepper and tomato seed lots produced under standard seed-industry conditions. Moreover, the paper shows the results of a systematic review of published data on seed transmission and outbreaks in commercial pepper and tomato crops. Based on the data of the grow-out trials and review of the literature, it is concluded that the role of seed transmission in the spread of pospiviroids in practice is possibly overestimated.

In another research paper, Bertacca et al. [11] develop a real-time loop-mediated isothermal amplification (LAMP) assay for simple, rapid, and efficient detection of the Olea europaea geminivirus (OEGV), a virus recently reported in different olive cultivation areas worldwide. This real-time LAMP assay results are also suitable for phytopathological laboratories with limited facilities and resources, as well as for direct OEGV detection in the field, representing a reliable method for rapid screening of olive plant material.

The research paper of Lukianova et al. [12] reports on the development of a specific and sensitive detection protocol based on a real-time PCR with a TaqMan probe for *Pectobacterium parmentieri*, a plant-pathogenic bacterium, recently attributed as a separate species and causing soft rot of potato tubers. Since no cross-reaction with the non-target bacterial species, or loss of sensitivity, is observed, this specific and sensitive diagnostic tool may reveal a wider distribution and host range for this bacterium, and will expand knowledge of the life cycle and environmental preferences of this pathogen.

In the research contribution of Shalaby et al. [13], the foliar application of paclobutrazol (PBZ) at different rates of 25, 50, and 100 mg L^–1^ enhances the quality of tomato seedlings and induces resistance to early blight (*Alternaria solani*) disease post inoculation at 7, 14, and 21 days under greenhouse conditions. Higher values in chlorophyll content, enzyme activities, cuticle thickness of stems and numbers, and thickness of stomata are recorded on PBZ-treated tomato plants.

In the research paper of Withee et al. [14], 16 isolates of *Paramyrothecium* spp. retrieved from 14 host species across nine plant families are collected and identified. In detail, a new species *Paramyrothecium vignicola* sp. nov. is identified according to morphological features and concatenated (ITS, cmdA, rpb2, and tub2) phylogeny. Further, *P. breviseta* and *P. foliicola* represent novel geographic records for Thailand, while *P. eichhorniae* represents a novel host record (*Psophocarpus* sp., *Centrosema* sp., *Aristolochia* sp.). These *Paramyrothecium* species fulfill Koch’s postulates and, moreover, cross pathogenicity assays on *Coffea arabica* L., *Commelina benghalensis* L., *Glycine max* (L.) Merr., and *Dieffenbachia seguine* (Jacq.) Schott reveal a potential multiple host range for these pathogens.

In another research paper, Alhudaib et al. [15] focus their attention on a devastating disease, i.e., the black scorch, for date palm plantations in Saudi Arabian Peninsula. The authors report variable symptoms as well as neck bending, leaf drying, tissue necrosis, wilting, and mortality of the entire tree in the Saudi Arabia peninsula. Based on morphological, molecular and pathogenicity assays, the causal agent of above mentioned symptoms is identified in *Thielaviopsis punctulata*. Further indications about the disease control are provided by authors who suggest using fosetyl Al- and difenoconazole-based formulates. These fungicides can be used in integrated disease management strategies to control black scorch disease.

Li et al. [16] develop in an interesting study a deep learning network called YOLO (You Only Look Once) for detecting jute diseases (-JD) from images. In the main architecture of YOLO-JD, the authors integrate three new modules, the Sand Clock Feature Extraction Module (SCFEM), the Deep Sand Clock Feature Extraction Module (DSCFEM), and the Spatial Pyramid Pooling Module (SPPM) to extract image features effectively. The authors also build a new large-scale image dataset for jute diseases and pests with ten classes. Compared with other state-of-the-art experiments, YOLO-JD achieves the best detection accuracy, with an average mAP (mean average precision) of 96.63%.

Aiello et al. [17] describe for the first time the new wilt symptoms on passion fruit caused by *Fusarium nirenbergiae*. This report also focuses on the phytopathological implications of this fungal pathogen, which may represent a future significant threat for the expanding passion fruit production in Italy and Europe.

In the last research paper, Calderone et al. [18] demonstrate that a chitosan oligosaccharide (COS–) oligogalacturonides (OGA) complex, applied alone or combined with arbuscular mycorrhizal fungi (AMF), is able to reduce significantly powdery mildew (*Erysiphe necator*), gray mold (*Botrytis cinerea*), and sour rot infections on red berried Nero d’Avola and white berried Inzolia wine grape cultivars. Overall, this strategy can be proposed as a valid and safer option for the sustainable management of the main grapevine pathogens in organic agroecosystems.
